# Refeeding Hypophosphatemia Among Critically Ill Surgical Patients: A Prospective Analysis of Incidence, Risk Factors, and Clinical Outcomes

**DOI:** 10.3390/nu18111655

**Published:** 2026-05-22

**Authors:** Tutkun Talih, Gamze Talih, Umut S. Eser, Kamile Eser, Gamze Gökçek, Dinçer Göksülük, Murat Sungur, Kürşat Gündoğan

**Affiliations:** 1 Department of General Surgery, Faculty of Medicine, Erciyes University, 38039 Kayseri, Turkey; tt3882@hotmail.com (T.T.); sercaneser@hotmail.com (U.S.E.); drgamzegokcek@gmail.com (G.G.); 2 Department of Anaesthesiology and Reanimation, Faculty of Medicine, Erciyes University, 38039 Kayseri, Turkey; 3 Department of Internal Medicine, Faculty of Medicine, Erciyes University, 38039 Kayseri, Turkey; kamiletatli@gmail.com; 4 Department of Biostatistics, Faculty of Medicine, Sakarya University, 54280 Sakarya, Turkey; dincergoksuluk@sakarya.edu.tr; 5 Department of Internal Medicine, Division of Intensive Care Medicine, Faculty of Medicine, Erciyes University, 38039 Kayseri, Turkey; msungur@erciyes.edu.tr (M.S.);; 6 Department of Clinical Nutrition, Health Sciences Institute, Erciyes University, 38039 Kayseri, Turkey

**Keywords:** critically ill surgical patients, mortality, outcome, refeeding hypophosphatemia, refeeding syndrome

## Abstract

**Background**: This study aimed to determine the incidence of refeeding hypophosphatemia (RH) in critically ill surgical patients admitted to the surgical intensive care unit, to identify associated risk factors, and to evaluate its impact on clinical outcomes. **Methods**: This prospective observational study included 135 patients admitted to the general surgery ICU for ≥48 h, with 109 included in the final analysis. Clinical, nutritional, and laboratory data from the first five ICU days were collected and evaluated. According to the baseline phosphorus level, a 10–20% decrease was classified as mild RH, a 20–30% decrease as moderate RH, and a decrease of more than 30% as severe RH. **Results**: Serum phosphorus levels over the first five days were 3.89 ± 1.5, 3.44 ± 1.6, 3.20 ± 1.6, 3.13 ± 1.7, and 3.35 ± 1.8 mg/dL, respectively, with the lowest level on Day 4. The overall RH incidence was 76% (11% mild, 11% moderate, 54% severe). In multivariable analysis, lower albumin, decreased HCO_3_ and higher WBC were associated with RH. Reoperation (18%) and shock (14%) were the most common complications. Mechanical ventilation was required in 62% of patients. Median ICU stay was 8 days, and ICU mortality was 22%. **Conclusion**: Refeeding hypophosphatemia is highly prevalent among critically ill surgical patients, with more than half of affected patients developing severe hypophosphatemia. Higher disease severity, hypoalbuminemia, and vasopressor use were identified as significant risk factors for RH.

## 1. Introduction

Refeeding syndrome (RFS) is a potentially life-threatening metabolic complication that may occur following the reintroduction of nutrition after prolonged fasting or malnutrition. It is characterized by electrolyte disturbances, particularly hypophosphatemia, together with fluid, metabolic, and organ dysfunction-related abnormalities. Among these abnormalities, hypophosphatemia is considered the earliest, most prominent, and clinically relevant biochemical manifestation of RFS and is frequently used as a surrogate marker in clinical studies [1,2].

According to the 2020 American Society for Parenteral and Enteral Nutrition (ASPEN) consensus recommendations, RFS is defined by a decrease in serum phosphorus, potassium, and/or magnesium levels occurring within five days after the initiation or substantial increase in nutritional support and may be accompanied by organ dysfunction and/or thiamine deficiency. ASPEN further classifies the severity of electrolyte decline as mild (10–20%), moderate (20–30%), or severe (>30%) [3,4]. Refeeding syndrome is characterized not only by electrolyte disturbances but also by clinical complications such as fluid overload, rhabdomyolysis, cardiopulmonary failure, seizures, encephalopathy, and coma. However, because the clinical manifestations of RFS are often nonspecific and may overlap with comorbid conditions in critically ill patients, many studies have focused primarily on refeeding hypophosphatemia (RH) as a more objective and measurable marker of refeeding-related metabolic disturbances [5].

Critically ill surgical patients may be particularly vulnerable to refeeding-related metabolic disturbances. Prolonged preoperative fasting, gastrointestinal dysfunction, impaired intestinal continuity, delayed enteral feeding due to hemodynamic instability, surgical stress, systemic inflammation, and malignancy-related malnutrition may all contribute to phosphate depletion and increase the risk of RH in this population [6]. In addition, surgical intensive care unit (ICU) patients are exposed to metabolic and physiological stressors that differ substantially from those observed in medical ICU populations.

Despite increasing awareness of RFS, its diagnosis remains challenging because there is still no universally accepted definition or diagnostic criterion. The reported incidence of RH and RFS varies widely across studies, partly due to heterogeneity in patient populations and differences in diagnostic approaches [7]. Furthermore, hypophosphatemia in critically ill patients may also develop secondary to factors unrelated to nutritional therapy, including respiratory alkalosis, vasopressor use, insulin administration, renal replacement therapies, and intracellular phosphate shifts associated with severe illness [5,8].

In the acute surgical setting, prioritization of hemodynamic stabilization and surgical success may further delay comprehensive nutritional assessment and monitoring. Early identification of RH may provide a critical window for intervention, allowing optimization of nutritional strategies and potentially preventing progression to clinically significant refeeding-related complications. However, despite its clinical relevance, data regarding the incidence, risk factors, and clinical outcomes of RH in surgical ICU patients remain limited.

Therefore, this study aimed to determine the incidence of RH in critically ill postoperative patients admitted to the surgical intensive care unit, to identify associated risk factors, and to evaluate its impact on clinical outcomes.

## 2. Materials and Methods

### 2.1. Study Design and Ethical Approval

This prospective observational study was approved by the Erciyes University Local Ethics Committee (approval date and decision number: 2025/183) and conducted in accordance with the Declaration of Helsinki. The patients were informed about the purpose of the study, and consent forms were signed.

### 2.2. Study Population

A total of 135 patients admitted to the general surgery ICU of our hospital for at least 48 h between May and September 2025 were screened. After applying the exclusion criteria, 109 patients were included in the final analysis. Twenty-six patients were excluded due to inadequate nutritional intake. Clinical, nutritional, and laboratory data collected during the first five days following ICU admission were evaluated.

Patients with a history of chronic kidney disease, psychiatric disorders, eating disorders, chronic alcohol or substance abuse, prior parathyroidectomy, previous treatment for hyperphosphatemia, or those who declined participation were excluded from the study.

### 2.3. Baseline Characteristics and Risk Assessment

Baseline demographic and clinical data including age, height, weight, body mass index (BMI), ICU admission diagnosis, Acute Physiology and Chronic Health Evaluation II (APACHE II) score, Sequential Organ Failure Assessment (SOFA) score, Nutrition Risk in the Critically Ill (NUTRIC) score, Global Leadership Initiative on Malnutrition (GLIM) criteria, and the Deyo–Charlson Comorbidity Index (CCI) were recorded at ICU admission.

The risk of RFS was assessed according to the National Institute for Health and Care Excellence (NICE) criteria. Patients were classified as having moderate or high risk for RFS, and NICE risk categories were documented for all patients [9].

### 2.4. Nutritional Management and Supportive Therapies

Daily caloric requirements were calculated as 20–25 kcal/kg/day according to current ICU nutrition guidelines [10]. For patients requiring oral nutrition, feeding was initiated once clinical conditions allowed adequate gastrointestinal function and safe oral intake. Oral feeding was progressively advanced based on swallowing capacity, gastrointestinal tolerance, and overall clinical stability. Enteral nutrition (EN) was administered to hemodynamically stable patients with a functioning gastrointestinal tract, while parenteral nutrition (PN) was used in patients with contraindications to EN or with inadequate enteral tolerance. Nutritional regimens were adjusted daily according to clinical status and metabolic requirements. Nutritional regimens for oral feeding were categorized according to the institutional surgical ICU protocol as Regimen I (clear liquid diet), Regimen II (full liquid/soft diet), and Regimen III (standard oral diet). Nutritional data included feeding modality, composition of nutritional products, routes of administration, macronutrient distribution (protein, fat, carbohydrate), non-nutritional caloric intake, and multivitamin supplementation.

Data regarding administered replacement fluids (5% or 10% dextrose solutions, Ringer’s lactate, Isolyte-S/M, and 0.9% normal saline), insulin therapy, electrolyte replacement (magnesium, potassium, phosphorus, calcium, sodium bicarbonate), and thiamine supplementation were recorded. Blood glucose levels were routinely monitored during ICU follow-up, and insulin therapy was initiated in patients with blood glucose levels > 180 mg/dL according to the institutional glycemic control protocol for critically ill patients. In patients considered at risk for refeeding syndrome, thiamine supplementation was administered at 300 mg/day per the institutional nutritional support protocol. Medication use, including antacids, diuretics, proton pump inhibitors, antidiabetic agents, and antihypertensive drugs, was also documented.

### 2.5. Laboratory and Clinical Monitoring

Laboratory parameters, including arterial blood gas analysis, complete blood count, serum electrolytes (sodium, potassium, magnesium, phosphorus, calcium), renal and liver function tests, and serum albumin levels, were measured at ICU admission and monitored daily during the first five days of ICU stay.

Blood product transfusions (erythrocyte suspension, fresh frozen plasma, and platelet concentrates) were recorded. Acute kidney injury (AKI) was defined according to the Kidney Disease: Improving Global Outcomes (KDIGO) criteria, and the need for continuous renal replacement therapy (CRRT) or intermittent hemodialysis was documented.

### 2.6. Definition of Refeeding Hypophosphatemia

Serum phosphorus levels were measured daily for the first five days following ICU admission. Baseline serum phosphorus was defined as the value obtained at ICU admission. RH was defined as a decrease in serum phosphorus concentration of more than 10% from baseline occurring within the first five days after the initiation or escalation of nutritional support. The severity of hypophosphatemia was classified as mild (10–20% decrease), moderate (20–30% decrease), or severe (>30% decrease). Patients who exhibited a decrease greater than 10% were considered to have developed RH [3].

Clinical and nutritional parameters were compared between patients who developed RH and those who did not.

### 2.7. Adverse Clinical Outcomes

Adverse clinical outcomes were prospectively recorded and included deterioration in oxygenation requiring supplemental oxygen (nasal cannula or face mask), high-flow oxygen therapy (HFOT), non-invasive mechanical ventilation (NIMV), or invasive mechanical ventilation (IMV); development of acute respiratory distress syndrome (ARDS); sepsis and/or shock; requirement for vasopressor or inotropic support; occurrence of arrhythmias and/or cardiac failure; reoperation; surgical site infection; and altered mental status.

Duration of mechanical ventilation, ICU length of stay, and in-ICU mortality were monitored and recorded.

### 2.8. Study Outcomes

The primary outcome of the study was the incidence of RH in patients admitted to the surgical ICU.

Secondary outcomes included the identification of risk factors for RH and its association with clinical outcomes.

### 2.9. Statistical Analysis

The sample size was planned a priori for multivariable logistic regression, since the main analytical objective of the study was to identify independent predictors of RH. Based on previous evidence and clinical expectation, the incidence of RH was assumed to be 70%. To ensure adequate model stability, a minimum of 10 outcome events per candidate predictor variable was targeted. Accordingly, at least 50 events would be required for a model including 5 candidate predictors, and at least 80 events for a model including 8 candidate predictors. Given an expected event proportion of 0.70, the corresponding minimum total sample size was estimated as 72 patients for a 5-predictor model and 115 patients for an 8-predictor model.

Statistical analyses were performed using SPSS version 23 (IBM Corp., Armonk, NY, USA). Continuous variables were first assessed for normality using the Shapiro–Wilk test and graphical approaches (e.g., Q-Q plots, histogram, etc.). Normally distributed data were expressed as mean ± standard deviation (SD) and compared using Student’s t-test, whereas non-normally distributed data were presented as median [interquartile range, Q1–Q3] and compared using the Mann–Whitney U test. Categorical variables were reported as counts and percentages (n, %) and analyzed using Chi-square or Fisher’s exact test when appropriate. A *p*-value < 0.05 was considered statistically significant.

To identify factors associated with RH, logistic regression analyses were conducted in two stages. First, each potential predictor was examined separately using univariable logistic regression. Variables considered clinically relevant and/or showing potential association in the univariable analyses were then entered into a multivariable logistic regression model. Variable selection for the multivariable model was performed using backward elimination based on the likelihood ratio test, with variables with *p*-values greater than 0.10 sequentially removed. The final model was formed from the remaining variables.

Regression results are presented as regression coefficients, standard errors, odds ratios, and 95% confidence intervals. Platelet and WBC values were rescaled by dividing by 1000 to improve interpretability. For categorical predictors, risk estimates were reported for the categories specified in parentheses. For albumin and HCO_3_, risk estimates and corresponding confidence intervals were expressed per one-unit decrease, since lower values were associated with increased risk of RH.

## 3. Results

**Figure 1:**
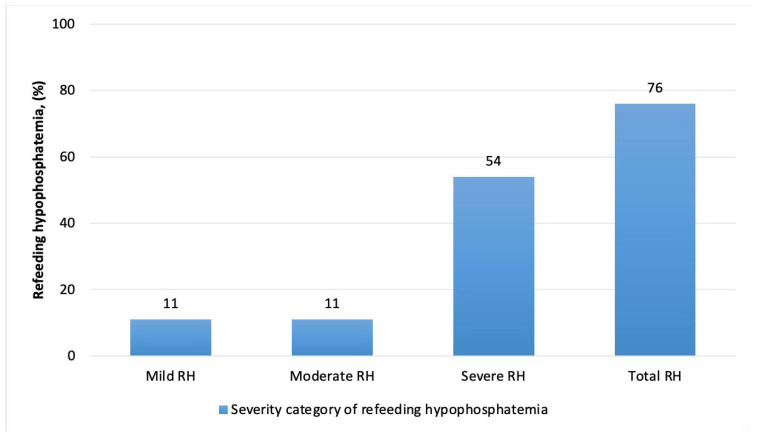
Frequency of RH in Surgical Critically Ill Patients.

A total of 109 critically ill surgical patients were analyzed. Most were male (64%), with a mean age of 62 years. RH developed in 83 patients (76%), of whom 58 (54%) had severe RH (Figure 1). Patients were divided into two groups based on the presence or absence of RH. Baseline characteristics, including age, sex, BMI, and comorbidity burden, are presented in Table 1, with no significant differences between the groups (*p* > 0.05 for all).

The RH (+) group had significantly higher APACHE II scores (18.33 ± 8.29 vs. 12.96 ± 8.31, *p* = 0.005), while other clinical and nutritional risk scores (NICE, NUTRIC, SOFA) were similar between groups. No differences were observed in malnutrition severity (GLIM) or ICU admission diagnoses (*p* > 0.05). Day 1 nutritional targets and caloric intake were also comparable; however, non-nutritional calorie intake was significantly higher in the RH (−) group (*p* = 0.019). Complication rates—including shock, reoperation, arrhythmia, surgical site infection, and respiratory failure—were compared between the groups, and no significant differences were observed (*p* > 0.05). Similarly, the need for mechanical ventilation, incidence of acute kidney injury, ICU length of stay, and mortality did not differ between the groups (*p* > 0.05). Notably, among patients requiring vasopressors, 93.5% developed RH whereas 6.5% did not, indicating a markedly imbalanced distribution of this variable between the groups (*p* = 0.007) (Table 1).

Acute kidney injury occurred in 31% of patients. Only three patients required CRRT, representing a very small proportion of the cohort, and therefore unlikely to have materially influenced the overall incidence of hypophosphatemia. RH (+) patients received thiamine supplementation more frequently than RH (−) patients (42% [n = 35] vs. 35% [n = 9], respectively; *p* = 0.493). Insulin therapy was administered more frequently in RH (+) patients compared to RH (−) patients (22% [n = 18] vs. 8% [n = 2], respectively; *p* = 0.108).

Patients’ feeding routes and nutrient intakes during the first five days in the ICU are summarized in Supplementary Table S1. On Day 1, 92% of patients received no oral or enteral feeding, while 8% received parenteral nutrition (PN). Mean daily energy intake increased from 731 kcal on Day 1 to a peak of 1097 kcal on Day 4, with a relatively stable macronutrient distribution (carbohydrates 39–42%, proteins 15–21%, lipids 39–42%). Non-nutritional energy intake increased after Day 2, peaking at 258 kcal on Day 3 and remaining elevated thereafter.

As shown in Table 2, most laboratory parameters were similar between RH (+) and RH (−) patients. However, patients with RH had significantly higher creatinine levels (*p* = 0.043). Patients with RH had significantly lower levels of calcium, total protein, and albumin compared to those without RH (*p* = 0.014, *p* = 0.008, and *p* = 0.004, respectively). In terms of acid–base status, the RH (+) group had significantly lower pH and bicarbonate levels (*p* = 0.002 and *p* < 0.001, respectively), indicating more pronounced metabolic acidosis; however, no significant differences were observed in partial pressures of oxygen and carbon dioxide or in lactate levels (*p* > 0.05). No significant differences were observed between the groups in blood glucose, BUN, electrolytes (sodium, potassium, chloride, magnesium), or hematological parameters (*p* > 0.05 for all variables).

**Figure 2:**
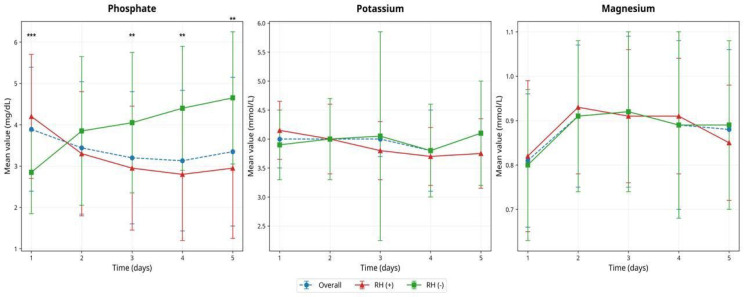
Changes in Serum Phosphate, Potassium and Magnesium Levels Over Time in Patients With and Without Refeeding Hypophosphatemia. ***; *p* < 0.001; **; *p* < 0.05.

Changes in serum phosphate, potassium, and magnesium levels are presented in Figure 2 and Table 3. Phosphate levels differed significantly between patients with and without RH on Days 1, 3, 4, and 5 (*p* < 0.05), whereas no significant differences were observed in magnesium and potassium levels between the groups. (*p* > 0.05).

**Figure 3:**
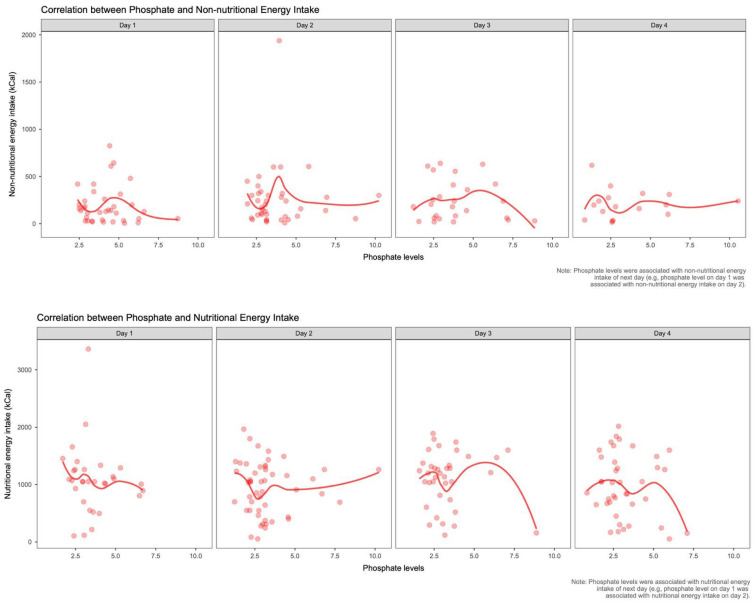
Correlation between phosphate nutritional and non-nutritional energy intake over days 1–4.

In the correlation analysis, serum phosphate levels were matched with the energy intake of the subsequent day; accordingly, phosphate measurements from days 1 to 4 were paired with the nutritional and non-nutritional energy intake variables of the following day. Spearman’s rank correlation analysis, performed separately for each day, showed no significant association between phosphate levels and either nutritional or non-nutritional energy intake. The observed relationships were visualized with scatter plots and LOESS trend curves (Table 4 and Figure 3).

Table 5 summarizes the findings from the univariable and multivariable logistic regression analyses for refeeding hypophosphatemia. In the univariable analyses, lower albumin, higher APACHE II score, lower HCO3, lower total protein, vasopressor requirement, lower calcium, and higher platelet count were significantly associated with refeeding hypophosphatemia at the 0.05 significance level. Potassium, WBC, and creatinine showed associations at the 0.10 level. In contrast, SOFA score, NUTRIC score, NICE high-risk status, and age were not significantly associated with refeeding hypo-phosphatemia.

In the multivariable logistic regression model, albumin, APACHE II score, HCO3, potassium, and WBC remained in the final model after backward elimination (Table 5). Lower albumin and lower HCO3 were independently associated with higher odds of refeeding hypophosphatemia. Each one-unit decrease in albumin was associated with a 3.06-fold increase in the odds of RH (OR: 3.06, 95% CI: 1.21 to 7.63, *p* = 0.018), while each one-unit decrease in HCO3 was associated with a 16% increase in the odds of RH (OR: 1.16, 95% CI: 1.03 to 1.33, *p* = 0.031). WBC was also independently associated with RH, with each 1000-unit increase corresponding to a 13% increase in the odds of RH (OR: 1.13, 95% CI: 1.02 to 1.25, *p* = 0.017).

APACHE II score and potassium were retained in the final model, but did not reach the conventional 0.05 significance level. Each one-point increase in APACHE II score was associated with a 5% increase in the odds of RH (OR: 1.05, 95% CI: 0.98 to 1.18, *p* = 0.168). Although this direction was consistent with clinical expectations, the association was not statistically significant. Potassium also showed a positive association with RH. This association was significant at the 0.10 level and may indicate a clinically relevant relationship (OR: 2.29, 95% CI: 0.81 to 6.56, *p* = 0.097). Only variables retained in the final multivariable model are presented in the multiple regression section of Table 5.

## 4. Discussion

In this study, the incidence of RH in critically ill surgical ICU patients was found to be 76%, with severe RH observed in 54% of cases. Identified risk factors for RH included lower albumin levels, decreased HCO_3_.

In many intensive care units, there are no established protocols for diagnosing and managing RH, and in some cases, clinicians may not even anticipate its development. A point-prevalence study demonstrated that approximately one-third of ICUs do not perform daily phosphate measurements [11]. The reported incidence of RFS ranges widely from 0% to 80%, largely due to the lack of a universally accepted definition and objective diagnostic criteria [12]. In an observational study conducted in China, the incidence of hypophosphatemia among critically ill ICU patients was reported as 77% [13].

Hypophosphatemia is the most prominent clinical manifestation of RFS. According to the 2020 ASPEN guidelines, RFS is characterized by a decline in serum phosphate, potassium, and/or magnesium levels following the reintroduction of nutrition and may be accompanied by organ dysfunction [3]. Many studies have also accepted hypophosphatemia or a rapid decline in serum phosphate levels from baseline as part of the RFS definition [14]. In a randomized controlled trial conducted by Doig et al. [15], RH was defined as either a decrease in phosphate levels greater than 0.16 mmol/L or a level below 0.65 mmol/L. Differences in patient populations and heterogeneity in definitions may explain the variability in reported RH incidence.

In the study by Fuentes et al. [1], the incidence of RH was 39% among critically ill surgical patients receiving enteral nutrition and 20% among those not receiving enteral nutrition. Consistent with our findings, RH was not associated with ICU length of stay or mechanical ventilation duration in that study. Additionally, Fuentes et al. reported no association between the initiation or adequacy of enteral nutrition and the development of RH. Current studies investigating RH in surgical ICU patients remain limited, often involving small sample sizes and retrospective designs [16,17]. In our cohort, a substantial proportion of patients (48% by day 5) received parenteral nutrition. This may be explained by gastrointestinal tract dysfunction and frequent intolerance to enteral feeding in surgical patients. Some studies suggest that patients receiving enteral nutrition may have a higher risk of developing RFS, potentially due to the incretin effect, which can rapidly increase insulin levels and contribute to electrolyte shifts [18]. It should also be noted that the definition of refeeding hypophosphatemia used in this study, based on a relative decrease in serum phosphate levels according to ASPEN consensus recommendations, may partly explain the high observed incidence. This definition allows the identification of early, clinically relevant phosphate shifts even in patients with normal absolute phosphate levels. However, such an approach may also capture transient ICU-related metabolic fluctuations, potentially leading to higher reported incidence rates than studies using absolute hypophosphatemia thresholds. Therefore, differences in diagnostic criteria across studies should be considered when interpreting the variability in reported RH incidence.

Marik and Bedigian [17] reported no difference in daily energy intake between patients who developed RH and those who did not in their prospective study. Similarly, in our study, neither nutritional nor non-nutritional energy intake was associated with phosphate levels. The high incidence of RH observed in our study may be related not only to nutritional intake but also to the characteristics of the surgical ICU population. Surgical critically ill patients are at increased risk of preoperative and early ICU nutritional insufficiency due to prolonged fasting periods, surgical stress, impaired gastrointestinal continuity, malabsorption, and underlying disease burden. In addition, the high proportion of patients undergoing cancer surgery in our cohort may have further contributed to baseline nutritional depletion. Therefore, preexisting malnutrition and critical illness-related metabolic stress may have contributed to the increased frequency of hypophosphatemia observed in our study. Furthermore, the absence of a significant association between nutritional intake and phosphate decline suggests that RH in this setting is likely multifactorial rather than solely nutrition-related. These findings should therefore be interpreted cautiously, particularly regarding causal relationships between feeding and changes in phosphate levels.

Although the NICE guidelines define risk factors for RFS [9], recent studies have shown that only about 30% of patients with RFS can be identified using these criteria due to their low sensitivity [19]. Therefore, additional risk factors beyond the NICE criteria may contribute to the development of RFS. A 2024 review reported that low albumin levels are associated with RFS. Evidence regarding the role of nutritional modality remains heterogeneous. While some studies have reported an association between enteral nutrition and RFS, others have found no such relationship. Similarly, conflicting results have been reported for parenteral nutrition. Malignancy has also been identified as a risk factor for RFS [18]. The high incidence of RH observed in our study may be related to the large proportion of patients undergoing cancer surgery and the presence of preoperative malnutrition. Our findings of low bicarbonate and albumin levels are consistent with previously reported risk factors. Additionally, low calcium and potassium have been identified in other studies as contributing factors.

Doig et al. [15] conducted the first randomized controlled trial to investigate whether energy restriction influences outcomes in critically ill patients with RFS. In this study involving 339 mechanically ventilated ICU patients, a full-calorie strategy following RFS diagnosis was associated with higher mortality at 60 and 90 days compared with a restricted-calorie approach.

In contrast, Coşkun et al. [20] reported higher mortality and longer ICU stays in patients with RFS in a retrospective study. However, this may be explained by the inclusion of a medical ICU population with a high prevalence of comorbidities (70%) and malignancy (20%). Olthof et al. [21] found no significant differences between patients with and without RFS in ICU and hospital length of stay, mechanical ventilation duration, or mortality. Furthermore, both Coşkun and Olthof reported no difference in mortality between patients with severe hypophosphatemia (<0.32 mmol/L ≈ <1.0 mg/dL) and those without. Variations in mortality risk across studies may partly be due to other causes of hypophosphatemia that confound the diagnosis of RFS.

Given the observational nature of this study, the identified associations should be interpreted cautiously and do not imply causality. The association between lower bicarbonate levels and RH may reflect the severity of critical illness rather than the effect of nutritional therapy alone. In critically ill surgical patients, metabolic acidosis is commonly associated with tissue hypoperfusion and organ dysfunction, both indicators of severe disease. In addition, factors such as surgical stress, inflammation, impaired gastrointestinal function, and intracellular electrolyte shifts may contribute to hypophosphatemia independent of classical refeeding mechanisms. Therefore, RH may represent not only refeeding-related metabolic changes but also the overall severity of illness in this population.

In our study, although hypophosphatemia was common in surgical ICU patients, no significant differences were observed between patients with and without RH in terms of mortality or ICU length of stay. Factors such as impaired gastrointestinal continuity, malabsorption, and surgical stress may have contributed to the development of hypophosphatemia independent of refeeding. These findings should be interpreted with caution due to the observational design, limited sample size, group imbalance, and relatively small number of mortality events. Accordingly, the results are exploratory and do not allow definitive conclusions regarding the impact of RH on clinical outcomes. Future studies with larger, multicenter cohorts are warranted to further evaluate the severity of hypophosphatemia.

### Limitations and Strengths

The results of this study should be interpreted with caution. The main limitations include the relatively small sample size and single-center design. A small number of patients received continuous renal replacement therapy. In addition, several factors affecting phosphate metabolism, including insulin dosing, transfusion burden, diuretic exposure, and cumulative phosphate replacement doses, could not be standardized or quantified comprehensively during the study period. Nevertheless, given the very limited number of patients receiving CRRT, its impact on the overall results is likely minimal. Limited availability of intravenous phosphate formulations in our country may also have affected the standardization of phosphate replacement practices. Furthermore, lower bicarbonate levels and metabolic acidosis observed in patients with RH may reflect greater illness severity rather than isolated refeeding-related metabolic alterations.

Another limitation of this study is the use of a relative decrease in serum phosphate levels to define refeeding hypophosphatemia, in accordance with ASPEN consensus criteria. While this approach is sensitive to early metabolic changes, it does not require absolute hypophosphatemia and may therefore overestimate the incidence of RH compared with definitions based solely on absolute phosphate thresholds. This methodological heterogeneity limits direct comparisons with studies that use different diagnostic criteria.

Strengths of the study include the prospective daily follow-up over five consecutive days and the detailed assessment of both nutritional and non-nutritional energy intake.

## 5. Conclusions

Refeeding hypophosphatemia is highly prevalent among critically ill surgical ICU patients, with a substantial proportion experiencing severe forms. Lower bicarbonate levels and hypoalbuminemia were identified as significant risk factors. These findings highlight the need for increased awareness, routine phosphate monitoring, and the development of standardized diagnostic and management protocols for refeeding syndrome in ICU settings. Further large-scale, multicenter studies are warranted to better define risk factors and optimize management strategies.

## Figures and Tables

**Table 1:** Demographic and clinical characteristics of surgical critically ill patients with refeeding hypophosphatemia.

Variables	All Participants N = 109	RH (+) N = 83	RH (−) N = 26	*p* Value
Age, ±SD (years)	62 ± 16	62 ± 16	64 ± 20	0.599
Gender, n (%) Male Female	70 (64) 39 (36)	55 (66) 28 (34)	15 (58) 11 (42)	0.426
BMI, ±SD	26.19 ± 5.36	26.4 ± 5.4	25.7 ± (5.7)	0.567
Charlson Co -Morbidite index, Median [Q1, Q3]	5 [3, 7.75]	5 [3, 8]	4 [2.7, 7]	0.268
APACHE II score, ±SD	17.05 ± 8.95	18.33 ± 8.29	12.96 ± 8.31	**0.005**
NICE score for RH, n (%) No risk High risk	59 (54) 50 (46)	42 (51) 41 (49)	17 (65) 9 (35)	0.187
NUTRIC score, [Q1, Q3]	4 [1, 5]	4 [2, 5]	3 [1, 5]	0.148
SOFA score (first day), [Q1, Q3]	5 [2, 8]	6 [2, 9]	4 [1.7, 6.2]	0.057
SOFA score (last day), [Q1, Q3]	2 [1, 5]	2 [2, 5]	2.5 [1, 6.3]	0.809
GLIM score, n (%) Stage 1 malnutrition Stage 2 malnutrition	31 (28) 25 (23)	26 (83.9) 18 (72.0)	5 (16.1) 7 (28.0)	0.282
Reason for ICU admission, n (%) Benign GI disorders GI malignancies Trauma Other	55 (50) 38 (35) 14 (13) 2 (2)	44 (80.0) 29 (76.3) 9 (64.3) 1	11 (20.0) 9 (23.7) 5 (35.7) 1	0.413
Target calories on day 1, ±SD (kcal)	1739 ± 306	1764 ± 304	1659 ± 307	0.126
Total nutritional calories received on day 1, ±SD (kcal)	731 ± 338	501 ± 238 (*n* = 6)	846 ± 334 (*n* = 3)	0.160
Non-nutritional calories on day 1, range (min-max) (kcal)	115 (12–613)	69 (27–101)	200 (80–290)	**0.019**
Complications during follow-up, n (%) Reoperation Shock Arrhythmia Wound infection Respiratory failure	20 15 8 5 4	14 (70.0) 13 (86.7) 7 (87.5) 3 (60.0) 2 (50.0)	6 (30.0) 2 (13.3) 1 (12.5) 2 (40.0) 2 (50.0)	0.411
Need for mechanical ventilation, n (%)	68 (62)	55 (81)	13 (19)	0.135
Need for vasopressor therapy, n (%)	31 (28.4)	29 (93.5)	2 (6.5)	**0.007**
Acute kidney injury, n (%)	34 (31)	27 (79.4)	7 (20.6)	0.590
Length of ICU stay, (min-max) (days)	8 (2–59)	9 (2–59)	7 (2–43)	0.436
ICU mortality, n (%)	24 (22)	17 (20)	7 (27)	0.489

*Values are presented as mean ± SD, median [Q1, Q3], or n (%). RH: refeeding hypophosphatemia; RH (+): developing group; RH (−): non-developing group. p < 0.05 was considered significant.*

**Table 2:** Baseline laboratory data of participants with and without refeeding hypophosphatemia.

Variables	All Patients N = 109	RH (+) N = 83	RH (−) N = 26	*p* Value
Blood glucose (mg/dL)	130 [102, 166]	171 [136, 232]	156 [119, 201]	0.242
BUN (mg/dL)	19.5 [13.3, 36.3]	19.5 [13.1, 37.9]	18.8 [14, 33.4]	0.688
Creatinine (mg/dL)	0.96 [0.69, 1.44]	0.99 [0.76, 1.45]	0.72 [0.57, 1.15]	0.043
Sodium (mmol/L)	139 [137, 142]	140 [137, 142]	139 [136.8, 141.3]	0.360
Potassium (mmol/L)	4.06 [3.72, 4.50]	4.08 [3.74, 4.57]	3.94 [3.47, 4.40]	0.101
Phosphate (mg/dL)	3.89 [2.91, 4.57]	2.86 [3.19, 4.69]	4.21 [2.23, 4.30]	<0.001
Chloride (mmol/L)	105 [102, 108.4]	105 [102, 109.3]	104 [102, 107]	0.278
Magnesium (mmol/L)	0.79 [0.69, 0.93]	0.79 [0.69, 0.93]	0.81 [0.70, 0.92]	0.983
Calcium (mg/dL)	7.72 [7.2, 8.2]	7.64 [7.1, 8.1]	8.15 [7.5, 8.5]	0.014
Total protein (g/dL)	4.82 [4.2, 5.4]	4.79 [4.1, 5.3]	5.19 [4.7, 6.0]	0.008
Albumin (g/dL)	2.67 [2.2, 3.1]	2.57 [2.2, 2.9]	2.92 [2.5, 3.7]	0.004
WBC (10^3^/µL)	11.2 [7.9, 16.7]	11.9 [7.9, 17.8]	9.9 [7, 12.9]	0.120
Hemoglobin (g/dL)	10.4 [9.4, 12.1]	10.3 [9.1, 12.1]	10.8 [9.5, 13.1]	0.373
Platelet (10^3^/µL)	223 [146, 326]	244 [149, 345]	180 [122, 282]	0.068
pH	7.38 [7.29, 7.44]	7.35 [7.27, 7.43]	7.42 [7.38, 7.46]	0.002
PaO_2_ (mmHg)	90.2 [73.1, 119.7]	92.1 [74.7, 125.4]	86 [67.5, 105.9]	0.291
PaCO_2_ (mmHg)	33.3 [29.9, 37.9]	33.1 [28.7, 37.9]	33.5 [32.1, 37.9]	0.607
HCO_3_ (mEq/L)	19 [16.6, 22]	18.1 [15.9, 20.8]	21.6 [19.3, 24.0]	**<0.001**
Lactate (mmol/L)	1.89 [1.37, 3.18]	1.86 [1.4, 3.4]	1.95 [1.3, 2.9]	0.777

*Values are presented as median [Q1, Q3]. RH: refeeding hypophosphatemia; RH (+): developing RH group; RH (−): non-developing RH group. Comparisons were made using the Mann–Whitney U test. p < 0.05 was considered statistically significant.*

**Table 3:** Serum Phosphate, Potassium, and Magnesium Levels Over Time in Patients With and Without Refeeding Hypophosphatemia.

	Day 1			Day 2			Day 3			Day 4			Day 5		
	RH (+)	RH (−)	*p*	RH(+)	RH(−)	*p*	RH(+)	RH(−)	*p*	RH(+)	RH(−)	*p*	RH(+)	RH(−)	*p*
**P (** **mg/dL)**	4.22 ± 1.45	2.86 ± 1.05	**<0.001**	3.31 ± 1.53	3.86 ± 1.84	0.136	2.95 ± 1.47	4.02 ± 1.75	**0.004**	2.80 ± 1.57	4.38 ± 1.47	**0.001**	2.97 ± 1.71	4.66 ± 1.64	**0.004**
**Mg** (**mmol/L)**	0.82 ± 0.17	0.80 ± 0.17	0.654	0.93 ± 0.15	0.91 ± 0.17	0.663	0.91 ± 0.15	0.92 ± 0.18	0.810	0.91 ± 0.13	0.89 ± 0.21	0.675	0.85 ± 0.13	0.89 ± 0.19	0.395
**K (mmol/L)**	4.16 ± 0.54	3.92 ± 0.58	0.053	4.00 ± 0.55	3.99 ± 0.67	0.953	3.80 ± 0.51	4.02 ± 1.75	0.13	3.72 ± 0.54	3.81 ± 0.79	0.632	3.76 ± 0.66	4.09 ± 0.94	0.186

**Table 4:** Correlation of phosphate levels with nutritional and non-nutritional energy intake.

Days	Energy Intake (kCal)	Rho	*p*-Value
1	Nutritional	−0.235	0.204
2	Nutritional	−0.084	0.561
3	Nutritional	−0.001	0.996
4	Nutritional	−0.113	0.489
1	Non-nutritional	−0.128	0.449
2	Non-nutritional	−0.085	0.604
3	Non-nutritional	−0.035	0.866
4	Non-nutritional	0.028	0.914

*Spearman’s correlation analysis was performed. The correlation coefficients given days were calculated against consecutive days.*

**Table 5:** Simple and multiple logistic regression results for the response “Refeeding Hypophosphatemia”.

	Simple			Multiple *		
Variable	Beta (SE)	OR (95% CI)	*p*	Beta (SE)	OR (95% CI)	*p*
Albumin ^†^	1.13 (0.37)	3.12 (1.51–6.25)	0.002	1.12 (0.47)	3.06 (1.21–7.63)	0.018
APACHE	0.09 (0.03)	1.09 (1.02–1.16)	0.007	0.05 (0.04)	1.05 (0.98–1.18)	0.168
HCO_3_ ^†^	0.16 (0.06)	1.16 (1.04–1.32)	0.007	0.15 (0.07)	1.16 (1.03–1.33)	0.031
Total Protein	−0.65 (0.26)	0.52 (0.32–0.86)	0.011			
Vasopressor (Yes)	1.86 (0.77)	6.44 (1.42–29.21)	0.016			
Calcium	−0.55 (0.27)	0.58 (0.34–0.98)	0.041			
Platelet (×1000)	0.004 (0.002)	1.00 (1.00–1.01)	0.043			
Potassium	0.84 (0.44)	2.31 (0.97–5.47)	0.057	0.83 (0.54)	2.29 (0.81–6.56)	0.097
WBC (×1000)	0.06 (0.04)	1.07 (0.99–1.15)	0.091	0.12 (0.05)	1.13 (1.02–1.25)	0.017
Creatinine	0.71 (0.43)	2.03 (0.87–4.74)	0.101			
SOFA	0.1 (0.06)	1.1 (0.98–1.25)	0.115			
NUTRIC	0.17 (0.11)	1.18 (0.95–1.47)	0.135			
NICE (High Risk)	0.61 (0.47)	1.84 (0.74–4.61)	0.191			
Age	−0.01 (0.01)	0.99 (0.97–1.02)	0.596			

**Note**: Platelet and WBC values were rescaled by dividing by 1000. Risk estimates were provided for the categories shown in parentheses for categorical predictors, namely vasopressor need and NICE. * Multiple logistic regression model with backward elimination variable selection. ^†^ For albumin and HCO3, risk estimates and corresponding confidence intervals are expressed per one-unit decrease, since lower values were associated with increased risk. Diagnostics: AUC = 0.81 [95% CI: 0.71 to 0.91]; VIFmax = 1.39; Brier Score = 0.134; Hoshmer–Lemeshow (p) = 0.426.

## Data Availability

The data presented in this study are available upon request from the corresponding author due to privacy restrictions.

## Supplementary Materials

The following supporting information can be downloaded at: https://www.mdpi.com/article/10.3390/nu18111655/s1, Table S1: ‘Nutritional data of surgical critically ill patients over five days’.

## References

[1] Fuentes E., Yeh D.D., Quraishi S.A., Johnson E.A., Kaafarani H., Lee J., King D.R., DeMoya M., Fagenholz P., Butler K. (2017). Hypophosphatemia in Enterally Fed Patients in the Surgical Intensive Care Unit. Nutr. Clin. Pract..

[2] Ergül S.Ş., Sahin G.G., Ozer N.T., Kaynar L., Celik S., Gundogan K. (2022). Evaluation of refeeding hypophosphatemia frequency, risk factors, and nutritional status during stem cell transplantation in patients with hematologic malignancy. Clin. Nutr. ESPEN.

[3] da Silva J.S.V., Seres D.S., Sabino K., Adams S.C., Berdahl G.J., Citty S.W., Cober M.P., Evans D.C., Greaves J.R., Gura K.M. (2020). Parenteral Nutrition Safety and Clinical Practice Committees, American Society for Parenteral and Enteral Nutrition. ASPEN Consensus Recommendations for Refeeding Syndrome. Nutr. Clin. Pract..

[4] Mehanna H.M., Moledina J., Travis J. (2008). Refeeding syndrome: What it is, and how to prevent and treat it. BMJ.

[5] Wong G.J.Y., Pang J.G.T., Li Y.Y., Lew C.C.H. (2021). Refeeding Hypophosphatemia in Patients Receiving Parenteral Nutrition: Prevalence, Risk Factors, and Predicting Its Occurrence. Nutr. Clin. Pract..

[6] Buitendag J., Variawa S., Davids R., Ahmed N. (2021). Refeeding syndrome in surgical patients post initiation of artificial feeding, a prospective cohort study in a low-income country. Clin. Nutr. ESPEN.

[7] Borriello R., Esposto G., Ainora M.E., Podagrosi G., Ferrone G., Mignini I., Galasso L., Gasbarrini A., Zocco M.A. (2025). Understanding Refeeding Syndrome in Critically Ill Patients: A Narrative Review. Nutrients.

[8] Kwiatkowska M., Krupnik D., Wesołek F., Jonczyk A., Krzych Ł. (2025). Comprehensive care of the patient with Refeeding Syndrome. Pol. Przegl. Chir..

[9] National Collaborating Centre for Acute Care (UK) (2006). Nutrition Support for Adults: Oral Nutrition Support, Enteral Tube Feeding and Parenteral Nutrition.

[10] Singer P., Blaser A.R., Berger M.M., Alhazzani W., Calder P.C., Casaer M.P., Hiesmayr M., Mayer K., Montejo J.C., Pichard C. (2019). ESPEN guideline on clinical nutrition in the intensive care unit. Clin. Nutr..

[11] Berger M.M., Appelberg O., Reintam-Blaser A., Ichai C., Joannes-Boyau O., Casaer M., Schaller S.J., Gunst J., Starkopf J. (2021). ESICM-MEN section. Prevalence of hypophosphatemia in the ICU—Results of an international one-day point prevalence survey. Clin. Nutr..

[12] Friedli N., Stanga Z., Sobotka L., Culkin A., Kondrup J., Laviano A., Mueller B., Schuetz P. (2017). Revisiting the refeeding syndrome: Results of a systematic review. Nutrition.

[13] Fu J.H., Zang B. (2012). The occurrence of hypophosphatemia and its prognostic value in intensive care unit patients. Zhongguo Wei Zhong Bing Ji Jiu Yi Xue.

[14] Doğan E., Gündoğan K., Temel Ş., Şahin S., Tuğra Özer N., Güneş Şahin G., Muhtaroğlu S., Sungur M., Güven M. (2021). The Association Between Refeeding Hypophosphatemia and Serum Appetite-Regulating Hormone Levels in Critically Ill Patients: A Prospective, Observational, Single-Center Pilot Study. J. Clin. Pract. Res..

[15] Doig G.S., Simpson F., Heighes P.T., Bellomo R., Chesher D., Caterson I.D., Reade M.C., Harrigan P.W., Refeeding Syndrome Trial Investigators Group (2015). Restricted versus continued standard caloric intake during the management of refeeding syndrome in critically ill adults: A randomized, parallel-group, multicentre, single-blind controlled trial. Lancet Respir. Med..

[16] Zazzo J.F., Troché G., Ruel P., Maintenant J. (1995). High incidence of hypophosphatemia in surgical intensive care patients: Efficacy of phosphorus therapy on myocardial function. Intensive Care Med..

[17] Marik P.E., Bedigian M.K. (1996). Refeeding hypophosphatemia in critically ill patients in an intensive care unit. A prospective study. Arch. Surg..

[18] Zheng P., Chen Y., Chen F., Zhou M., Xie C. (2025). Risk factors for the development of refeeding syndrome in adults: A systematic review. Nutr. Clin. Pract..

[19] Xiong R., Huang H., Wu Y., Wang S., Wang D., Ji Z., Lin Z., Zang N., Pan S., Huang K. (2021). Incidence and outcome of refeeding syndrome in neurocritically ill patients. Clin. Nutr..

[20] Coşkun R., Gündoğan K., Baldane S., Güven M., Sungur M. (2014). Refeeding hypophosphatemia: A potentially fatal danger in the intensive care unit. Turk. J. Med. Sci..

[21] Olthof L.E., Koekkoek W.A.C.K., van Setten C., Kars J.C.N., van Blokland D., van Zanten A.R.H. (2018). Impact of caloric intake in critically ill patients with, and without, refeeding syndrome: A retrospective study. Clin. Nutr..

