# Supplementary material for: Refeeding Hypophosphatemia Among Critically Ill Surgical Patients: A Prospective Analysis of Incidence, Risk Factors, and Clinical Outcomes
Source: Nutrients. 2026 May 22;18(11):1655. doi: 10.3390/nu18111655 (PMC13258305; doi:10.3390/nu18111655)
Supplement: Supplementary file 1 [file nutrients-18-01655-s001.zip › nutrients-4295374-supplementary.pdf]

Supplementary Table S1: Nutritional data of surgical critically ill patients over five days

Variables	Day 1	Day 2	Day 3	Day 4	Day 5
**Route of feeding, n(%)**					
None	100 (92)	78 (72)	55 (54)	30 (45)	17 (31)
Oral	0	7 (6)	13 (13)	9 (13)	8 (15)
EN Tube feeding	0	0	0	1 (2)	3 (6)
Parenteral Nutrition	9 (8)	24 (22)	34 (33)	27 (40)	26 (48)
PN+Oral	0	0	0	0	0
**Type of parenteral nutrition n(%)**					
Perifeic	9 (82)	16 (59)	18 (43)	15 (45)	10 (35)
Santral	2 (18)	11 (41)	24 (57)	18 (55)	19 (65)
Patients received energy, range (mean (min-max)). calorie	731.39 (315-1268)	1074.10 (106-3360)	935.36 (54-1966)	1096.97 (119-1890)	973.58 (54-2016)
Patients received carbohydrate ratio, %	42	40	41	39	41
Patients received protein ratio, %	15	21	20	19	20
Patients received lipid ratio, %	42	39	40	41	40
Patients received non-nutritional energy, range (mean (min-max)), calorie	115.30 (12-613)	119.25 (5-825)	257.93 (10-1938)	245.89 (20-640)	209.77 (16-619)

Footnote: Daily nutritional intake and feeding routes in critically ill surgical patients over the first five ICU days, including oral feeding, enteral nutrition (EN), and parenteral nutrition (PN). Data are presented as numbers (percentages) or mean values with ranges where appropriate.
